# Phenotypic and genotypic characterization of Human Immunodeficiency Virus type 1 CRF07_BC strains circulating in the Xinjiang Province of China

**DOI:** 10.1186/1742-4690-6-45

**Published:** 2009-05-14

**Authors:** Liying Ma, Yanfang Guo, Lin Yuan, Yang Huang, Jianping Sun, Shuiling Qu, Xiaoling Yu, Zhefeng Meng, Xiang He, Shibo Jiang, Yiming Shao

**Affiliations:** 1 State Key Laboratory for Infectious Disease Control and Prevention, National Center for AIDS/STD Control and Prevention, Chinese Center for Disease Control and Prevention, Beijing 100050, PR China; 2 Department of Pediatrics, the First Affiliated Hospital of Xinjiang Medical University, Urumqi 830054, PR China; 3 School of Pharmaceutical Sciences, Southern Medical University, Guangzhou 510515, PR China; 4 Lindsley F Kimball Research Institute, New York Blood Center, New York, NY 10021, USA

## Abstract

**Background:**

HIV-1 CRF07_BC recombinant previously circulated mainly among the intravenous drug users (IDUs) in Xinjiang province of China and is currently spreading in the entire country. The aim of this study is to characterize the genotypic and phenotypic properties of HIV-1 CRF07_BC isolates in comparison with those of the subtype B' (Thailand B) which is prevalent in the former plasma donors (FPDs) in China.

**Results:**

Twelve HIV-1 CRF07_BC variants were isolated from the blood of the HIV-1-infected IDUs in Xinjiang province, and 20 subtype B' isolates were obtained from the FPDs in Anhui and Shanxi provinces of China. All the CRF07_BC viruses utilized CCR5 co-receptor, whereas 12 subtype B' viruses were R5-tropic, and the remaining B' isolates were dual (R5X4) tropic. CRF07_BC viruses had lower net charge value in the V3 loop and exhibited slower replication kinetics than subtype B' viruses. The number and location of the potential N-linked glycosylation sites in V1/V2 and the C2 region of the CRF07_BC viruses were significantly different from those of the subtype B' viruses.

**Conclusion:**

The HIV-1 CRF07_BC recombinant strains with relatively lower net charges in the V3 loop exclusively utilize CCR5 co-receptor for infection and exhibit slow replication kinetics in the primary target cells, suggesting that CRF07_BC may be superior over B' and other HIV-1 subtypes in initiating infection in high-risk population. These findings have molecular implications for the adaptive evolution of HIV-1 circulating in China and the design of tailored therapeutic strategy for treatment of HIV-1 CRF07_BC infection.

## Background

The human immunodeficiency virus type 1 (HIV-1) which has high genetic diversity is classified into groups M, N, and O. The group M viruses that are responsible for the global AIDS epidemic have been further categorized into nine HIV-1 genetic subtypes – A, B, C, D, F, G, H, J, and K, as well as more than 34 circulating recombinant forms (CRFs) [1]. The recombinants may have enhanced fitness over their parental strains, resulting in increased pathogenicity [2,3]. In addition, a high prevalence of intersubtype recombinants (ISR) has also been reported in some areas [4].

In late 1980s, initial HIV-1 epidemic among intravenous drug users (IDUs) in Yunnan province, in the southwest of China, was caused by a mixture of subtype B and Thai subtype B (B'), but the B' subtype became dominate in the middle of the 1990s [5]. At the same time, subtype C viruses from India were also circulating among the IDUs, causing another HIV-1 epidemic in that region [6]. Due to the co-existence of the subtypes B' and C, some CRFs of HIV-1, e.g. CRF07_BC and CRF08_BC, formed and gradually predominated among the IDUs in Yunnan and Guangxi provinces, in southern China [7]. The appearance of CRF forms of HIV-1 in China indicates that the viruses are evolving dynamically [8]. Interestingly, the subtype B' viruses spread from Yunnan province to Henan, Hubei, Anhui, and Shanxi provinces, all located in central China, among the former plasma donors (FPDs), while the CRF07_BC viruses spread among the IDUs along the drug-trafficking routes to Xinjiang province, in the northwest of China [9]. CRF07_BC was reported to be responsible for more than 90% of the new HIV-1 infections in Xinjiang province [10]. Subsequently, CRF07_BC has become one of the most commonly transmitted HIV-1 subtypes across the country [6]. The latest national molecular epidemiology survey (2001–2003) showed that the prevalence of the HIV-1 CRF_BC has reached over 50%, compared with 30% in the first survey (1996–1998); whereas the prevalence of HIV-1 B' subtype showed a decrease from 48% in the first survey to 32% in the second survey, due to the improvement of blood safety [11].

The present study aims to characterize the genotype and phenotype of HIV-1 CRF07_BC strains circulating in Xinjiang province, in comparison with those of the subtype B' predominating in Anhui and Shanxi provinces. In doing so, we hope to provide information for understanding the adaptive evolution of HIV-1 CRF07_BC which could assist in the choosing of proper antiretroviral therapy regimens for treating patients infected by HIV-1 stains that are predominantly circulating in China.

## Results

### Sample population

**Figure 1:**
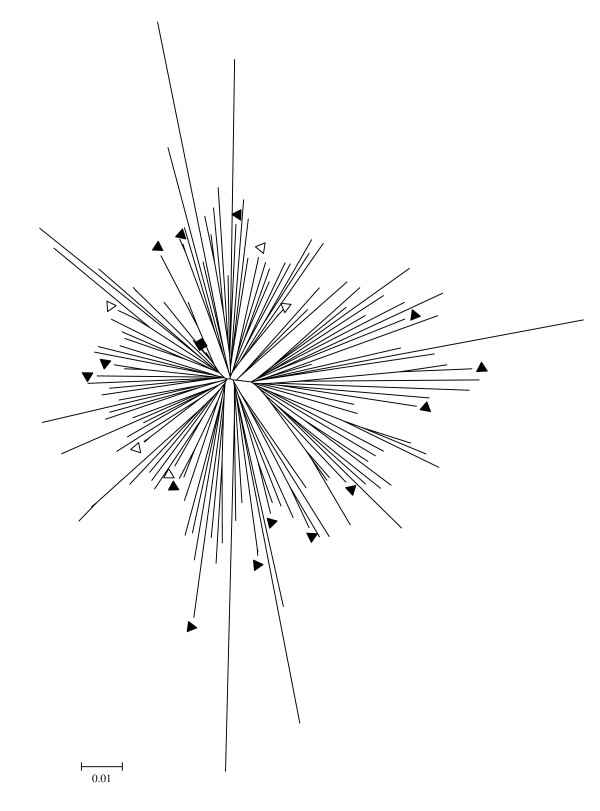
**Neighbor-joining genetic analysis of the phylogenetic tree of the HIV-1 CRF07_BC isolates**. The viral *env *sequences were obtained by PCR analysis of plasma samples of 124 HIV-1 infected patients, from whom the study subjects were selected.**"**black square" represents the consensus *env *sequence of CRF07_BC strains circulating in China. Blood samples were collected from the HIV-1-infected patients and used for isolation of the CRF07_BC isolates with (black triangle) or without (open triangle) *in vitro *infectivity.

The HIV-1 CRF07_BC and B' isolates were obtained from the blood of pre-selected HIV-1-infected patients, who participated in a multicenter AIDS Cohort Study in China during 2003–2005. All patients signed an individual informed consent form before blood collection. This study was approved by the Institutional Research Ethics Committee of Chinese Center for Disease Control and Prevention in China. To obtain the representative CRF07_BC isolates, we conducted Neighbor-joining genetic analysis CRF07_BC *env *sequences obtained from the plasma samples of 124 HIV-1-infected patients using PCR technique as previously described [10]. The phylogenetic tree was then constructed (Fig. 1). We selected 19 representative sequences of the viruses without epidemic link and collected blood samples from the patients who were infected by the corresponding viruses. From the cultures of peripheral blood mononuclear cells (PBMCs) in these blood samples, we successfully isolated 14 viruses with *in vitro *infectivity, but excluded two of them from this study because these two viruses were obtained from the patients who had used antiretroviral therapeutics (ART) before. All the patients are intravenous drug users (IDUs) from Xinjiang province of China. In a similar way, we obtained 20 representative subtype B' isolates without epidemical linkage from the blood samples of HIV-1-infected patients who were former plasma donors (FPDs) from Shanxi province (n = 3) and Anhui Province (n = 17) of China and who have not experienced ART before. The average age of the subjects was 36 (range: 27 – 49) years old. 10 out of 12 (83.3%) patients infected by CRF07_BC had a CD4 count > 200/μl, and 3 of them (25%) had a viral load < 10^4 ^copies/ml. By contrast, only 7 out of 20 (35%) patients infected by subtype B' virus showed a CD4 count > 200/μl, and none of them (0%) had a viral load < 10^4 ^copies/ml (Table 1).

**Table 1:** Geographic locations, sources of infection, CD4 counts and viral loads in the blood of the patients infected by the HIV-1 CRF07_BC and sub-type B'

Patients (#code)	Location (province)	infected via*	CD4 (copies/mL)	Viral load count/μL	HIV-1 sub-type
XJDC1353	Xinjiang	IDU	341	<LDL	CRF07_BC
XJDC6371	Xinjiang	IDU	590	3.549E+05	CRF07_BC
XJDC0793	Xinjiang	IDU	291	2.192E+06	CRF07_BC
XJDC0015	Xinjiang	IDU	322	3.40E+04	CRF07_BC
CBJB105	Xinjiang	IDU	463	2.312E+05	CRF07_BC
XJDC6431	Xinjiang	IDU	298	1.427E+05	CRF07_BC
XJN0135	Xinjiang	IDU	588	<LDL	CRF07_BC
CBJB257	Xinjiang	IDU	376	8.982E+03	CRF07_BC
CBJB256	Xinjiang	IDU	75	1.000E+06	CRF07_BC
XJDC1981	Xinjiang	IDU	237	5.482E+05	CRF07_BC
XJDC6331	Xinjiang	IDU	319	1.470E+06	CRF07_BC
XJDC6291	Xinjiang	IDU	155	1.18E+06	CRF07_BC
SHXDC168	Shanxi	FPD	6	2.91E+07	B'
SHXDC162	Shanxi	FPD	30	3.38E+06	B'
SHXDC148	Shanxi	FPD	11	2.32E+06	B'
20100311	Anhui	FPD	484	6.12E+04	B'
20201188	Anhui	FPD	61	3.24E+05	B'
20101324	Anhui	FPD	128	6.44E+04	B'
20101810	Anhui	FPD	196	3.45E+05	B'
20100374	Anhui	FPD	48	5.46E+05	B'
20101796	Anhui	FPD	125	5.77.E+05	B'
20100141	Anhui	FPD	16	6.33E+05	B'
20100419	Anhui	FPD	57	1.48E+05	B'
20200407	Anhui	FPD	41	1.17E+05	B'
20200084	Anhui	FPD	139	1.87E+05	B'
20100687	Anhui	FPD	264	3.55E+04	B'
20200068	Anhui	FPD	387	1.60E+04	B'
20200092	Anhui	FPD	365	4.21E+04	B'
20200259	Anhui	FPD	479	2.58E+05	B'
20200079	Anhui	FPD	221	1.25E+05	B'
20200108	Anhui	FPD	246	1.22E+04	B'
20100096	Anhui	FPD	141	2.86E+04	B'

*FPD: former plasma donation; IDU: intravenous drug use.

### Genotypic characterization of the CRF07_BC gp120

HIV-1 gp120 sequences from 12 CRF07_BC and 20 subtype B' viruses were compared for their differences in the number of positively charged amino acid residues in V3 loops, in the glycosylation variations in V3 loops, and in the potential N-linked glycosylations in other variable loops.

### The CRF07_BC viruses have lower net charge value in the gp120 V3 loops than those from the subtype B' viruses

**Figure 2:**
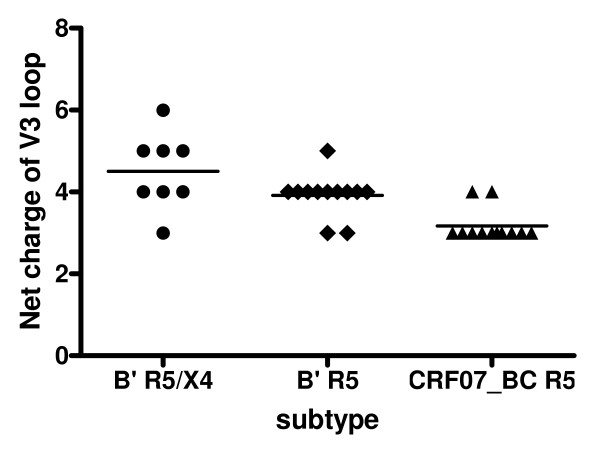
**Comparison of the net charge of V3 loops between CRF07_BC and subtype B' viruses**. The net charge of the V3 loop of CRF07_BC virus (3.17 ± 0.39) is significantly lower than both of the subtype B' R5/X4 virus (4.5 ± 0.93, *P *= 0.0003) and subtype B' R5 virus (3.92 ± 0.51, *P *= 0.0006), but there is no difference of the net charge of the V3 loop between subtype B' R5/X4 and R5 group.

The net charge value of the V3 loop in gp120 of the CRF07_BC and subtype B' viruses was calculated by subtracting the number of the negatively charged amino acids [aspartic acid (D) and glutamic acid (E)] from the number of positively charged amino acids [arginine (R) and lysine (K)]. Among the 12 CRF07_BC viruses, all had the GPGQ motif in the V3 loop. No positively charged amino acid residues were found at positions 11 and 25. The net charge value of the V3 loop ranged from 3 to 4 (3.17 ± 0.39), which is in agreement with our previous report [12]. In the 20 subtype B' isolates, 5 had GPGK (25%), 2 had GPGQ (10%), and 13 had GPGR (65%) in their V3 loops. There were four and one positively charged amino acids at position 11 and 25, respectively. The net charge value of the V3 loop was in a range from 3 to 6 (4.5 ± 0.93) for R5/X4 virus and in a range from 3 to 5 (3.92 ± 0.51) for R5 virus (Table 2). Overall, the net charge value of the V3 loop of CRF07_BC virus was significantly lower than that in the subtype B' R5/X4 virus (*P *= 0.0003) and R5 virus (*P *= 0.0006). There was no significant difference in the net charge value of the V3 loop between subtype B' R5/X4 and R5 viruses (Fig. 2).

**Table 2:** Comparison of the tropism (co-receptor usage), net charges and sequences of the gp120 V3 loops of CRF07_BC and sub-type B' viruses

HIV-1	Subtype	Tro-	V3 sequence*	
			
isolate		pism	Net charge	11 tip 25
XJDC1353	CRF07_BC	R5	4	CIRP*NNNTR *K **S**VRI~~GPGQ TFYATG**D**IIG DIRKAYC
XJDC6371	CRF07_BC	R5	4	CTRP*NNNTR *K **S**IRI~~GPGQ TFYATG**E**IIG NIRQAYC
XJDC0793	CRF07_BC	R5	3	CTRP*NNNTR *K **S**IRI~~GPGQ TFYATG**E**IIG DIRQAHC
XJDC0015	CRF07_BC	R5	3	CTRP*NNNTR *K **S**IRI~~GPGQ TFYATG**D**IIG DIRQAHC
CBJB105	CRF07_BC	R5	3	CTRP*NNNTR *K **S**IRI~~GPGQ TFYATG**E**IIG DIRQAHC
XJDC6431	CRF07_BC	R5	3	CTRP*NNNTR *K **S**IRI~~GPGQ TFYATG**E**IIG DIRQAYC
XJN0135	CRF07_BC	R5	3	CTRP*NNNTR *K **S**IRI~~GPGQ TFYATG**D**IIG DIRQAHC
CBJB257	CRF07_BC	R5	3	CTRP*NNNTR *K **S**IRI~~GPGQ AFYATG**D**IIG DIRQAHC
CBJB256	CRF07_BC	R5	3	CTRP*NNNTR *K **S**IRI~~GPGQ TFYATG**E**VIG DIRQAFC
XJDC6331	CRF07_BC	R5	3	CTRP*NNNTR *K **S**IRI~~GPGQ TFYAHG**E**IIG DIRQAYC
XJDC6291	CRF07_BC	R5	3	CTRPGNNTRK **S**IRI~~GPGQ TFYATG**D**IIG DIRQAHC
XJDC1981	CRF07_BC	R5	3	CTRP*NNNTR *K **S**IRI~~GPGQ TFYATG**D**IIG DIRQAHC
SHXDC148	B'	R5	4	CTRPTTNTRK **S**IPL~~GPGR AWYATG**P**IIG DIRQAHC
SHXDC168	B'	R5/X4	4	CTRP*NNNTR *K **S**INI~~GPGQ ALYATG**Q**IIG NIRQAHC
SHXDC162	B'	R5/X4	4	CTRP*NNNTR *K **S**IP.~~GPGR AWYTTG**Q**IIG DIRQAHC
20100311	B'	R5/X4	5	CTRP*NNNTR *K **R**VTL~~GPGR VWYTTG**Q**IVG DIRQAHC
20201188	B'	R5/X4	5	CTRP*NNNTR *N **R**FSI~~GPGR AWIATR**Q**IIG DIRQAHC
20101324	B'	R5/X4	5	CTRP*NNNTR *K **R**VTL~~GPGR VWYTTG**Q**IIG DIRKAHC
20101810	B'	R5/X4	4	CTRP*NNNTR *K **S**INL~~GPGR AWYTTG**Q**IF. DIRQAHC
20100374	B'	R5/X4	6	CTRP*NNNTR *K **R**VTL~~GPGR VWYTTG**Q**IIG DVRRAHC
20101796	B'	R5/X4	3	CTRPNNNTIK **S**ISL~~GPGK AWYTTG**Q**IIG DIRQAHC
20100141	B'	R5	4	CTRP*NNNTR *K **S**IPI~~GPGR AWYATG**Q**IIG DIRQAHC
20100419	B'	R5	4	CTRP*NNNTR *K **S**IHL~~GPGR AWFATG**E**IIG NIRQAHC
20200407	B'	R5	5	CTRPNNNTSK **G**IRI~~GPGR AWYATE**R**IVG DIRQAHC
20200084	B'	R5	3	CIRP*NNNTR *K **S**ITL~~GPGK AWYTTG**E**IIG DIRQAHC
20100687	B'	R5	4	CIRP*NNNTR *K **S**IHL~~GPGK AWYTTG**Q**IIG DIRQAHC
20200068	B'	R5	3	CIRP*NNNTR *K **S**IHL~~GPGQ AWYTTG**Q**IIG DIRQAHC
20200092	B'	R5	4	CTRP*NNNTR *K **S**IPL~~GPGK AWYTTG**Q**IIG EIRQAHC
20200259	B'	R5	4	CTRP*NNNTR *K **S**IPL~~GPGK AWYTTG**Q**IIG DIRQAHC
20200079	B'	R5	4	CTRP*NNNTR *K **G**IPL~~GPGR AWYATG**Q**IIG DIRQAHC
20200108	B'	R5	4	CTRP*NNNTR *K **S**INL~~GPGR AWYATG**Q**IIG EIRQAHC
20100096	B'	R5	4	CTRP*NNNTR *K **S**IHL~~GPGR AWYTTG**Q**IIG DIRQAHC

*The residues at the positions 11 and 25 in V3 loop were marked in bold, and those at the V3 tip were labeled with underline. The NNNTR motif was highlighted in *Italic*.

### There is no significant difference in the frequency of the potential N-linked glycosylation sites in the gp120 V3 loop between CRF07_BC and subtype B' viruses

The major glycosylation site, NNT, in the V3 loop (N301) was found in all 12 CRF07_BC viruses and in 19 of the 20 subtype B' viruses (Table 2). These results indicated that there is no difference in the V3 loop glycosylation sites between CRF07_BC and B' subtype viruses (*P *> 0.05).

### There is a significant difference in the number and location of potential N-linked glycosylation sites in C2 and V1/V2 regions of gp120 between CRF07_BC and subtype B' viruses

**Figure 3:**
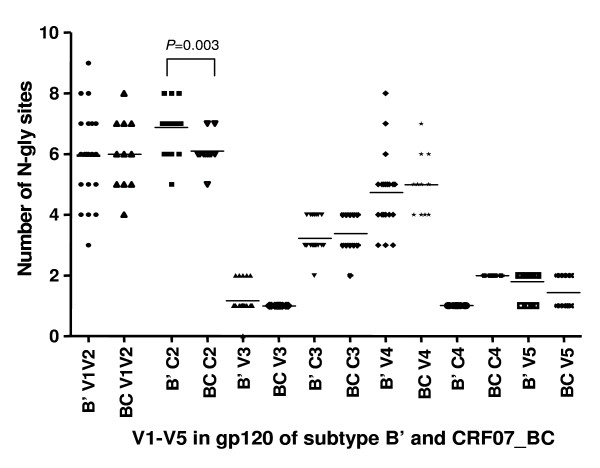
**The frequency of potential N-linked glycosylation sites in gp120 of the CRF07_BC and subtype B' viruses**. Both CRF07_ BC and subtype B' have N-linked glycosylation sites in the gp120 V1–V5 loops. There is no significant difference in the frequency of the N-linked glycosylation sites in V1–V5 loop except in C2 region (*P *= 0.003) between CRF07_BC and subtype B' virus.

**Figure 4:**
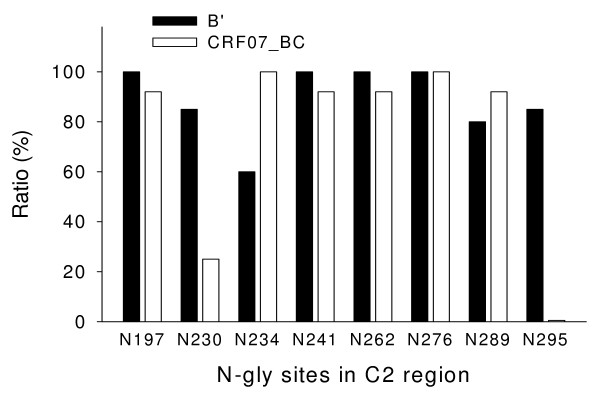
**The frequency of N- potential N-linked in the C2 region in gp120 of the CRF07_BC and subtype B' viruses**. There are significant differences in the frequency of potential N-linked glycosylation sites at the positions of N230 (*P *= 0.035), N234 (*P *= 0.015) and N295 (*P *< 0.001) between CRF07_BC and subtype B' virus.

**Figure 5:**
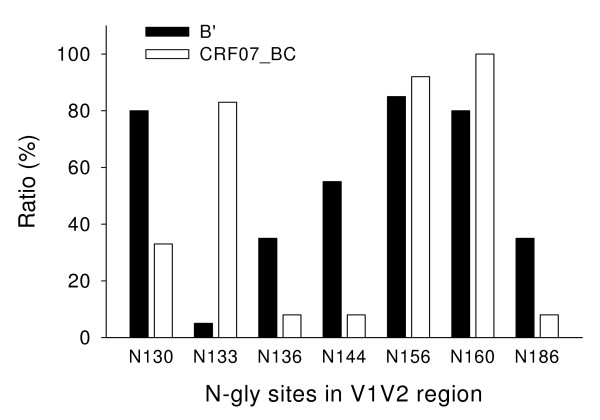
**The frequency of potential N-linked glycosylation sites in the V1V2 region in gp120 of the CRF07_BC and subtype B' viruses**. There is a significant difference in the frequency of N-linked glycosylation sites, including N130, N133, N136, N144, and N186 in the V1/V2 region between the CRF07_BC and subtype B' viruses.

The number and location of the potential N-linked glycosylation sites in the V1–V5 regions in gp120 of the CRF07_ BC and subtype B' viruses were analyzed. The results showed that the frequency of the potential N-glycosylation sites in the C2 region, particularly at the positions of N230, N234 and N295, was significantly different (*P *= 0.003) between CRF07_BC and subtype B' viruses (Fig. 3 and 4). There was also a significant difference in the frequency of N-glycosylation sites, including N130, N133, N136, N144, and N186 in the V1/V2 region between the CRF07_BC and subtype B' viruses (Fig. 5)

### Characterization of the CRF07_BC phenotype – CRF07_BC viruses exclusively utilize CCR5 co-receptor for infection while subtype B' viruses are R5-tropic or dual-tropic

Using GHOST cell-based assay, we detected the co-receptor usage of the CRF07_BC and subtype B' viruses. We found all 12 CRF07_BC viruses used the CCR5 co-receptor for infection, while 8 out of the 20 subtype B' isolates were dual-tropic (R5/X4-tropic), and the remaining B' viruses were R5-tropic. None of the viruses exclusively used the CXCR4 co-receptor for infection (Table 2).

### There is no significant difference in the infectivity between CRF07_BC and subtype B' viruses

**Figure 6:**
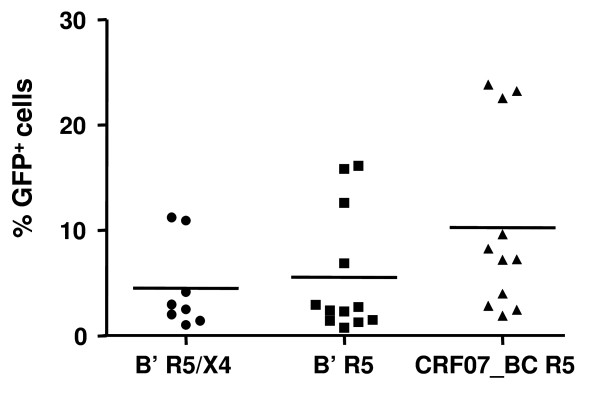
**Comparison of the infectivity of CRF07_BC R5 viruses with that of the subtype B' R5X4 and R5 viruses**. The infectivity of CRF07_BC strains (10.3% GFP^+ ^cells on average) was slightly higher than that of subtype B' with R5/X4 (5% GFP^+ ^cells on average) and R5 viruses (6% GFP^+ ^cells on average), but there was no significant difference among these three groups.

The infectivity of the CRF07_BC and subtype B' viruses were compared using a single-cycle infectivity assay with GHOST cells expressing CCR5 or CXCR4 as previously described [13]. As shown in Fig. 6, the infectivity of CRF07_BC strains (mean 10.3% GFP^+ ^cells) was slightly higher than that of subtype B' with R5/X4 (mean 5% GFP^+ ^cells) and R5 viruses (mean 6% GFP^+ ^cells), but there was no significant difference among these three groups.

### HIV-1 CRF07_BC viruses have slower replication kinetics than subtype B' viruses

**Figure 7:**
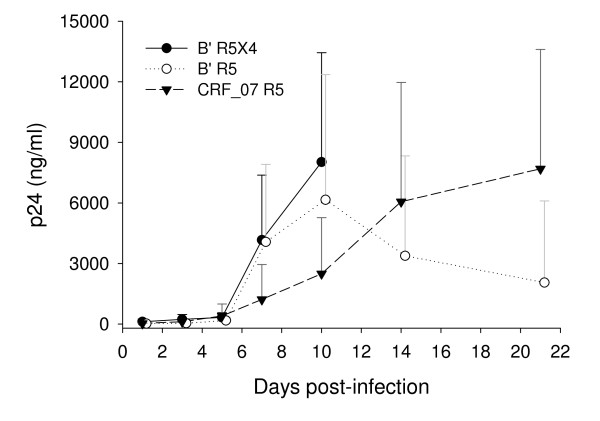
**Comparison of the replication kinetics of the CRF07_BC R5 virus with that of the subtype B' R5 and R5/X4 viruses**. The viral replication was determined by ELISA for p24 production. Each sample was tested in duplicate using the PBMCs from the same healthy blood donor. The experiment was repeated using the PBMCs from another healthy blood donor. The data are presented in mean ± SD.

The replication kinetics of CRF07_BC and subtype B' viruses were analyzed in PBMC cultures. The same viral input from each isolate was added to the PHA-activated PBMCs from healthy blood donors. The culture supernatants were collected for detection of p24 production on days 1, 3, 5, 7, 10, 14, and 21 days post-infection. But for subtype B' R5X4 virus, no further collection of the culture supernatants was done after 10 days of viral infection because the replication of this virus at its peak time resulted in significant cytopathic effect (CPE) on the PBMCs in the culture. As shown in Fig. 7, the replication kinetic of CRF07_BC isolates (peaking at day 21) was significantly slower than that of subtype B' isolates (peaking at day 7).

## Discussion

HIV-1 enters its target cell through a series of steps, including the interaction between the viral envelope glycoprotein (Env) surface subunit gp120 with the CD4 molecule and a chemokine co-receptor (CCR5 or CXCR4) on the target cell, and the subsequent conformational change of the *Env *transmembrane subunit gp41 [14]. The viruses using CCR5 and CXCR4 are designated R5 and X4, respectively [15]. HIV-1 co-receptor usage is associated with viral tropism, pathogenesis, and disease progression because viruses that utilize CCR5 (R5) initiate infections, while viruses that use CXCR4 (X4) emerge later in HIV-1 infected individuals to herald accelerated disease progression. The molecular alterations associated with the R5-to-X4 switch of CRF07_BC recombinant viruses *in vivo *and their biological manifestations have been reported[16].

In the present study, we found that all 12 HIV-1 isolates from the blood of IDUs were CRF07_BC R5 viruses, including those from patients with low CD4 counts. In contrast, all 20 isolates from the blood of FPDs were subtype B' viruses, including 12 R5 and 8 dual-tropic (R5X4) viruses. The net positive charge value of V3 loop in the gp120 of CRF07_BC was significantly lower than that in the subtype B' R5 and R5X4 viruses. It is well known that the net positive charge of the V3 loop plays a critical role in determining viral co-receptor tropism and pathogenesis. The V3 loops of R5-tropic viruses generally have a lower net positive charge than those of X4 [17-19]. The introduction of a few of positively charged residues (e.g. Arg) in the V3 results in the switch of the viral co-receptor tropism from R5 to X4 [20], and this switch from R5 to X4 tropism has been associated with more rapid clinical progression to AIDS. Furthermore, the net positive charge of the V3 loop also plays a key role in the immunological escape and co-receptor tropism evolution of HIV-1 *in vivo *because the viruses with less net positive charges in their V3 loop become more resistant to the anti-V3 neutralizing antibodies [21]. This selective force is continuously enriching the R5 viruses during long-lasting persistent infection. The relatively low net charge in the V3 loop of the CRF07_BC strain may contribute to its R5 tropism for infection of new target cells that express CD4 and CCR5.

Proper N-linked glycosylation is important for correct folding and function of viral envelope glycoproteins [22,23]. Alternation of the frequency of N-linked glycosylation sites at various locations in gp120 may significantly affect its function (e.g. receptor binding and mediating membrane fusion), and its antigenicity and immunogenicity. Here we found that there was no significant difference in the frequency of the *NNNTR *motif, which is associated with the co-receptor switch [24] in V3 loop between CRF07_BC and B' strains (Table 2). However, the number of the potential N-linked glycosylation sites in the gp120 C2 region of the subtype B' strains is significantly higher than that in the same region of the CRF07_BC strains (Fig. 2). Particularly, the frequency of N230 in the B' strains is remarkably higher than that in the CRF07_BC strains. However, the frequency of N234 in the B' strains is lower than that in the CRF07_BC strains (Fig. 3). Notably, more than 80% of the B' strains show the N295 glycosylation site, while this site was absent in all CRF07_BC strains (Fig. 3). This observation is consistent with the report by Khan et al. [25] who found that N295 was absent in the HIV-1 subtype C isolates from India. Loss of N295 may affect CD4 binding since the N448-linked G2 glycan is flanked on each side by N295-linked and N262-linked glycans, and these three glycans protrude to form a cluster of sugar recognized by the CD4 molecule. Therefore, loss of the N295-linked glycan may result in disruption of the binding site for CD4 [26]. It has been reported that 2G12, a broadly neutralizing human mAb that specifically binds to a carbohydrate-dependent epitope on gp120, is generally ineffective against HIV-1 subtype C isolates [27]. The absence of an N-linked glycan at position 295 is correlated with resistance to 2G12 because replacement of N295 with alanine resulted in a significant decrease in 2G12 binding affinity to gp120 [28]. This suggests that the HIV-1 B' and CRF07_BC strains may have different sensitivity to the neutralizing antibodies that recognize the carbohydrate-dependent epitopes on gp120.

There is also a significant difference in the number of N-linked glycosylation sites in the V1V2 regions of the gp120 between the HIV-1 B' and CRF07_BC strains. The frequency of potential N-linked glycosylation sites in the gp120 V1V2 regions (N130, N136, N144, and N186) of the HIV-1 B' strains is significantly higher than that in the same regions of the CRF07_BC strains, but the frequency of N133 in B' strains is markedly lower than that in CRF07_BC strains (Fig. 4). These results suggest a difference in the number and location of the potential N-linked glycosylation sites in the V1/V2 regions of gp120 between the HIV-1 CRF07_BC and B' strains which may be relevant to their co-receptor usage. Previous studies have shown the potential N-linked glycosylation sites in the V1/V2 regions and those proximal to the V3 loops of gp120 were functionally critical because the carbohydrate moieties in these regions play essential roles in retaining the appropriate conformation of the variable loops for optimal interaction with the receptor and co-receptors [29]. Mutations in or near V1/V2 may compensate for the deleterious V3 mutations and may need to precede V3 mutations to permit virus survival.

Although no significant difference in the infectivity between CRF07_BC and subtype B' viruses was observed in this study, the replication kinetic of CRF07_BC variants is slower than that of subtype B' viruses. The relatively low replication kinetic of CRF07_BC viruses may not be attributed to its R5-tropism because both subtype B' R5 and R5X4 viruses have faster replication kinetics than that of CRF07_BC. It is unclear whether the replication kinetics and co-receptor usage of CRF07_BC are associated with its virulence since a majority (10 out of 12) of the patients infected by CRF07_BC participated in this study had a CD4 count > 200/μl, while only 7 out of 20 patients infected with subtype B' showed a CD4 count > 200/μl. Further study is warranted to investigate whether the variable frequency of the N-linked glycosylation sties in V1V2 and C2 region may contribute to the difference in the co-receptor usage and the replication kinetics between CRF07_BC and subtype B' viruses.

In conclusion, this study, for the first time, characterizes the genotypic and phenotypic properties of HIV-1 CRF07_BC strains circulating in Xinjiang province of China, in comparison with those of the HIV-1 subtype B'. The HIV-1 CRF07_BC viruses have lower net charge in V3 loop of gp120, exclusively utilize CCR5 co-receptor for infection, and exhibit slower replication kinetics than the subtype B' viruses. This study thus provides important information for understanding the molecular evolution of the Env sequences of the HIV-1 strains circulating in different geographic regions in China. This understanding could assist in the rational design of appropriate therapeutic regimens to treat HIV-1-infected patients in the corresponding regions in China.

## Methods

### Virus isolation

Peripheral blood mononuclear cells (PBMCs) were isolated from blood of the HIV-1-infected patients and healthy donors using Ficoll-Paque gradient (Amersham Biosciences; Uppsala, Sweden). The patients' PBMCs were co-cultured with phytohemagglutinin (PHA)-stimulated PBMCs from healthy donors. The cell cultures were maintained for 4 weeks in RPMI 1640 medium (Gibco) containing 20 U/ml of recombinant interleukin-2 (IL-2), 1% penicillin and streptomycin (P/S), 2 mM glutamine and 10% FBS and the culture media were changed twice a week. The culture supernatants were collected for detection of HIV-1 p24 production using a commercial enzyme-linked immunosorbent assay (ELISA) kit (Vironostika HIV-1 Microelisa system; BioMérieux; Marcy l'Etoile, France) and those containing p24 antigen > 1 ng/ml were aliquoted and stored in liquid nitrogen until used [30].

### Sequence analysis of the HIV-1 Env gp120

DNA was extracted from PBMCs infected by isolated viruses using a DNA blood Mini Kit (QIAGEN; Hilden, Germany). The sequences of the gp120 region were amplified by a nested polymerase chain reaction (nest-PCR) using the Gene Amp PCR System 9700 (Applied Biosystems, Foster City, California, USA) with the external primers ED5/ED12 (5'-ATGGGATCAAAGCCTAAAGCCATGTG-3' and 5'-AGTGCTTCCT GCTGCTCC CA-3') at 94°C, 3 min; 50°C, 1 min; and 72°C, 1.5 min for 3 cycles; then 94°C, 15 s; 50°C, 45 s; and 72°C, 1 min for 32 cycles, followed by 72°C for 10 min for a final extension. The second round PCR was performed with the internal primers ENV7/ENV8 (5'-CTGTTAAATGGCAGTCTAGC-3'and 5'-CACTTCTCCAATTGTCCCTCA-3') at 94°C, 2 min; 55°C, 45 s; and 72°C, 1.5 min for 1 cycle; then 94°C, 30 s; 55°C, 30 s; and 72°C, 1 min for 30 cycles, followed by 72°C for 10 min for a final extension. PCR products were identified on an agarose gel by electrophoresis, purified (Gel Extraction Kit, QIAGEN), sequenced on an ABI 377 Sequencer (Applied Biosciences), and analyzed using GCG Sequence Analysis software [31,32].

### Detection of viral load (VL) and CD4^+ ^cell count

Plasma VL was measured using an HIV-1 nucleotide fluorescence quantitative assay kit (BD Biosciences, Franklin Lakes, NJ, USA) with a lower detection limit (LDL) of 500 copies/ml. CD4^+ ^cell counts were assessed by FACS analysis with the FACS/Lyse kit provided by BD Biosciences [33].

### Assays for virus co-receptor usage and assessment of infectivity

GHOST cells that express CD4 and a chemokine receptor CCR5 or CXCR4 were used for measuring the co-receptor usage of the isolated viruses. The GHOST-R5 and GHOST-X4 cells (for R5 and X4 viruses, respectively) were seeded in 24-well plates (Corning Incorporated, Spain) at 6 × 10^4 ^cells/well/0.5 ml of Dulbecco's modified Eagle's medium (DMEM) (HyClone; Logan, Utah, USA) supplemented with 10% FBS, 1% L-glutamine, 1% penicillin plus streptomycin, Geneticin (500 μg/ml), hygromycin (100 μg/ml) and puromycin (1 μg/ml). On the following day, the medium was removed, and the monolayers (about 70% confluent) were infected with virus stocks (200 μl/well) in the presence of 8 μg/ml DEAE-dextran to enhance infection efficiency. After 16–18 h incubation in a 37°C and 5% CO_2 _humidified environment, virus and DEAE-dextran were replaced with 1 ml media. Cells were harvested 4 days post-infection and the cell monolayers were washed once again with PBS, re-suspended in 300 μl of 1 mM EDTA in PBS, and fixed in paraformaldehyde at a final concentration of 2%. GFP expression was then analyzed by flow cytometry (Elite ESP; Beckman Coulter). For each test, the GHOST-R5 and -X4 cells infected with or without SF33 were used as positive or negative controls, respectively. The infectivity of primary viral isolates was assessed in a single-cycle infectivity assay with GHOST-R5/X4 cells as described by Bleiber et al. [34]. Briefly, 4 × 10^4 ^cells were infected in duplicate with 2 ng of p24 antigen equivalent of virus. A positive control and negative control were included in each experiment to assess inter-assay variation and cell autofluorescence. The infectivity of the primary HIV-1 isolates was quantified based on the proportion of GFP-expressing cells determined by fluorescence-activated cell sorting at 24 h post-infection [35].

### Determination of viral replication kinetics

Replication kinetics of the HIV-1 CRF07_BC and B' isolates were compared. Briefly, each of the viral isolates was inoculated with an equal viral input (2 ng p24) into 5 × 10^6 ^PHA-stimulated PBMCs obtained from HIV-seronegative blood donors. After incubation at 37°C overnight, the cells were washed and re-suspended in complete medium supplemented with recombinant interleukin-2. The cultures were maintained for three weeks and the culture media were changed twice a week. Culture supernatants were collected every two to three days for measuring p24 antigen production by using ELISA kits (Coulter Beckman)[30].

### Statistical analysis

The gp120 V3 loop positive charges of the different subtypes were expressed as mean ± standard deviation (SD). Student's t test was used in the statistical analysis. The difference in glycosylation between subtype B' and CRF07_BC viruses was compared using the χ^2 ^test (Fisher's exact probability). All *P *values are two-sided, and a *P *value of <0.05 was considered significant. All statistical analyses were performed with the SPSS10.0 software.

## Competing interests

The authors declare that they have no competing interests.

## Authors' contributions

LM and SJ designed the study, analyzed the data, and drafted the manuscript. YG, YL, JS, SQ, XY, ZM, YH, XH collected samples and performed the experiments. YS supervised and directed the studies. All authors read and approved the final manuscript.
